# Efficient conditional knockout targeting vector construction using co-selection BAC recombineering (CoSBR)

**DOI:** 10.1093/nar/gkv600

**Published:** 2015-06-18

**Authors:** Robert J. Newman, Merone Roose-Girma, Søren Warming

**Affiliations:** Genentech, Inc., Department of Molecular Biology, 1 DNA Way, South San Francisco, CA 94080, USA

## Abstract

A simple and efficient strategy for Bacterial Artificial Chromosome (BAC) recombineering based on co-selection is described. We show that it is possible to efficiently modify two positions of a BAC simultaneously by co-transformation of a single-stranded DNA oligo and a double-stranded selection cassette. The use of co-selection BAC recombineering reduces the DNA manipulation needed to make a conditional knockout gene targeting vector to only two steps: a single round of BAC modification followed by a retrieval step.

## INTRODUCTION

Due to convenience of use and the ability to precisely engineer even large pieces of DNA, recombinogenic engineering or ‘recombineering’ (1–3) has become the standard method for producing vectors for gene targeting by homologous recombination in mouse embryonic stem (ES) cells. When the goal is to knock out a gene of interest in a tissue- or cell-type specific manner, a conditional knockout (CKO) allele is required. A typical design for a CKO allele is to flank (or ‘flox’) a critical exon(s) by two loxP sites, allowing for subsequent Cre-mediated recombination and thus removal of the floxed exon(s), resulting in a knockout (KO) allele. Typically, a CKO allele is designed so that the remaining exons after Cre-mediated recombination would splice out of frame, to ensure complete KO of the gene.

Several protocols for the production of conditional KO targeting vectors have been published (4,5). Current recombineering methods for construction of vectors for conditional KO alleles all essentially consist of four steps (5): (i) Retrieval of the desired genomic piece from a bacterial artificial chromosome (BAC) clone into a plasmid; (ii) precise insertion of a loxP-flanked bacterial selection cassette into a desired position upstream of a critical exon(s); (iii) removal of the selection marker by Cre-mediated recombination in *Escherichia coli*, leaving a single loxP site and (iv) insertion of an FRT-neo-FRT-loxP dual host selection cassette downstream of the critical exon(s) for positive selection in bacteria as well as ES cells. After ES cell targeting, the neo cassette can be removed by Flp expression, resulting in the CKO allele. The region between the two loxP sites can then be removed by systemic or cell type-specific Cre expression, resulting in a KO allele.

Our laboratory has used this approach to produce numerous CKO targeting vectors, and while this method has proven reliable and efficient, the actual number of DNA manipulations is larger than the four steps outlined above due to the need to separate engineered plasmid from parental plasmid: after each plasmid recombineering step a mixture of targeted and un-targeted plasmid in the same clone is almost always observed (6,7), indicating that parental and targeted plasmids are physically linked and thus cannot be separated by random segregation. As part of the recombineering process, introduced and processed single-stranded linear fragments serve to prime Okazaki fragment synthesis during replication, resulting in half of the molecules being modified and the other half being the un-modified original plasmid (8,9). The apparent lack of separation of the two plasmid species by segregation is explained by the fact that upon inhibition of the *E. Coli* RecBCD exonuclease by lambda prophage-encoded Gam, pBR322-derived plasmids (such as pUC and pBluescript, commonly used vectors) replicate by rolling-circle mode, resulting in the generation of large multi-unit plasmid molecules ((10), further discussed in (6) and (7)). After RecBCD exonuclease is re-activated, the multimeric plasmid is replicated as one large molecule containing both parental and targeted plasmid ‘repeats’. The two plasmid species therefore have to be separated by linearization followed by self-ligation and re-transformation. By selecting for only the recombined plasmid that contains the introduced selection marker, the parental plasmid is eliminated. While not technically difficult to perform after each round of recombineering, these extra steps add significant time to the construction process. To eliminate one round of digestion and self-ligation, the order of the two first steps can be reversed so the BAC is targeted with the loxP-flanked selection cassette prior to retrieval. Performing all the recombineering steps in the BAC has the potential to reduce the overall number of manipulations needed and thus greatly speed up the vector construction process. A method for high-throughput construction of CKO vectors by recombineering in the BAC has been described (11). However, this method also requires a ligation step and therefore does not take full advantage of BAC recombineering.

An efficient method for multiplex recombineering of the bacterial genome by co-selection multiplex automated genome engineering (CoS-MAGE) has been described (12,13). CoS-MAGE likely works by enriching for other, non-selectable, events in relative proximity to a selectable locus: In bacteria that have been modified at a selectable locus, the replication fork must have been available for modification at that locus, and it is therefore possible that other, proximal, loci could have been modified at the same time as well, when the replication fork was in the open state. Although not referred to specifically as CoS-MAGE, the ability to obtain modifications at more than one bacterial locus simultaneously has been reported by others as well (14,15).

We wanted to test if the principle of co-selection recombineering could be applied to BAC engineering as well, reducing the number of recombineering steps needed. In our hands both BAC recombineering and retrieval always result in pure clones, containing either parental or targeted species, but never both. Therefore, performing the genomic modification in the BAC, followed by a retrieval step, would completely eliminate the need for physical separation of any plasmid species.

Here, we describe the development of co-selection BAC recombineering, dubbed ‘CoSBR’, as well as a highly efficient two-step method for CKO vector generation using this approach.

## MATERIALS AND METHODS

A detailed protocol for generation of CKO vectors using CoSBR is supplied as supplemental information.

### Conditional targeting vector design

The vectors were designed to flank a critical exon(s) of a target gene with loxP sites to allow for conditional removal by Cre recombinase. Our design and choice of critical exons(s) ensures that splicing of the remaining exons after Cre-mediated removal of the critical exon(s) results in frame-shifts and premature stop codons, leading to a functional null allele. The VISTA browser (http://pipeline.lbl.gov/cgi-bin/gateway2) was used to guide the design of the 5′ and 3′ homology arms and the two loxP insertion sites, thus taking into account information about evolutionary conserved regions as well as repetitive elements.

### Custom DNA synthesis

All custom DNA synthesis was done by Blue Heron Biotechnology/Origene (Bothell, WA, USA). Retrieval arms were synthesized and subsequently cloned by Blue Heron into our retrieval vector pBlight-TK. *Pgk1*-em7-neo-ready cassettes were synthesized and cloned into Blue Heron's standard pUC vector.

### BAC clones

C57BL/6J BAC clones (RPCI-23 library, pBACe3.6 vector) for each gene were identified using the BAC End Pairs track on the UCSC genome browser ((16) http://genome.ucsc.edu/) and the Mouse July 2007, NCBI37/mm9, assembly (17). BAC clones were obtained from Life Technologies (Thermo Fisher Scientific, Inc). Replication of BACs is initiated at OriS and the replication fork moves unidirectionally in the same direction as the Sp6 promoter is transcribed (from the XhoI site in the OriS toward the BglII site 130 bp downstream (18), see Supplementary Figure S1). The lagging strand can be determined by the orientation of the genomic insert in the following way: Using the BAC end sequence track on the UCSC genome browser, orientation of the genomic insert in the BAC vector backbone is indicated by arrows in the direction from T7 toward Sp6. Replication of the BAC is in the opposite direction of these arrows (Sp6 toward T7) and the lagging strand (5′ to 3′) therefore has the same direction as the arrows. If the gene of interest is transcribed in the same direction as the T7 promoter in the BAC, then the lagging strand is equal to the coding strand of the gene. If the gene of interest is transcribed in the opposite direction as the T7 promoter, then the lagging strand is equal to the non-coding (template) strand (see Supplementary Figure S1).

### Preparation of the targeting cassette and retrieval plasmid

**Figure 1.**
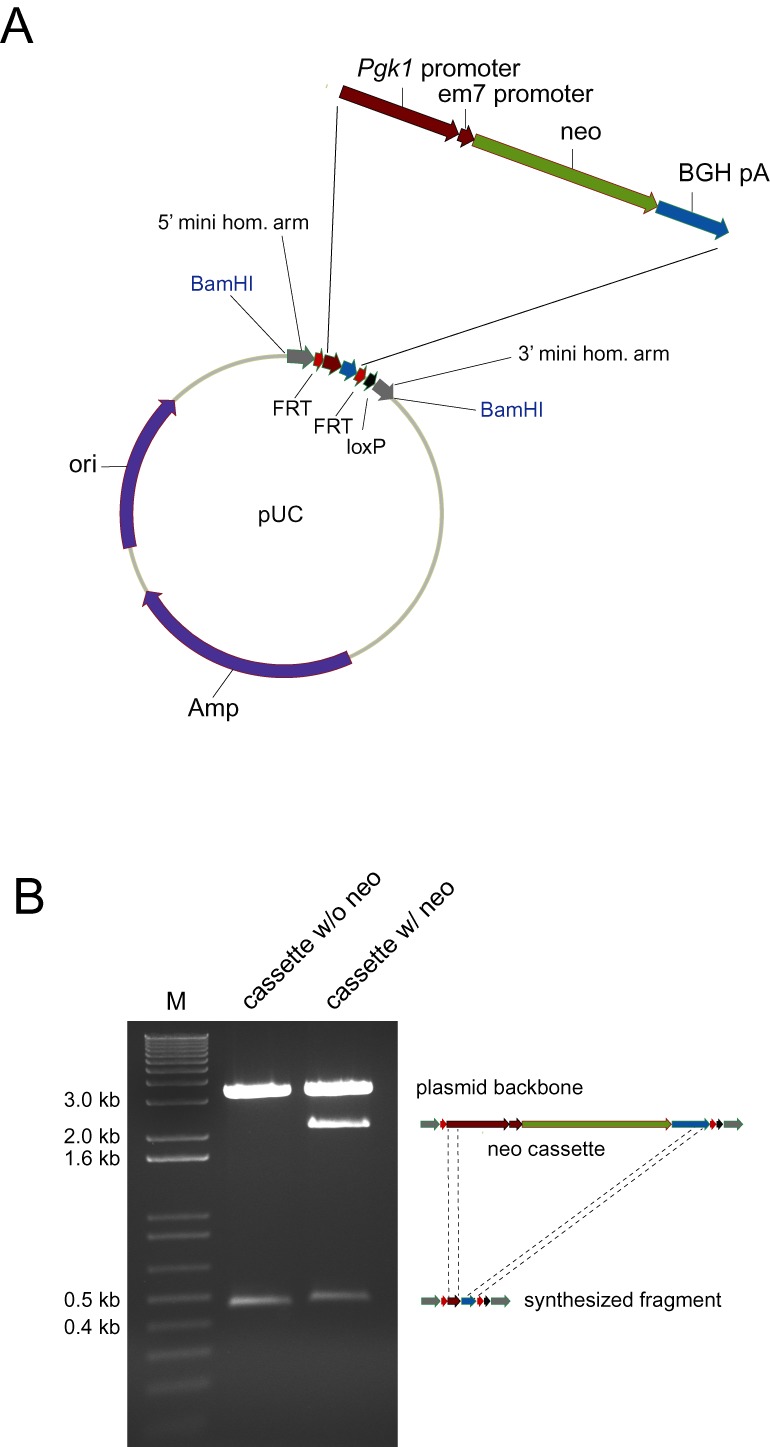
Strategy for generating a BAC targeting neo cassette by gene synthesis and recombineering. (**A**) A small sequence is synthesized and cloned into a standard pUC plasmid. The insert contains a 5′ mini homology arm flanking the future insertion site of the selection cassette, an FRT site, homology to the 5′ end of the mouse *Pgk1* promoter, homology to the 3′ end of the bovine growth hormone (BGH) polyadenylation cassette, another FRT site, a loxP site and a 3′ homology arm flanking the future cassette insertion site. An EcoRI-BamHI fragment from PL452 containing the entire dual eukaryotic/prokaryotic neo cassette is introduced into the target plasmid by recombineering. Precise integration into the plasmid is achieved by recombination with the synthesized *Pgk1* and BGH pA homology fragments. (**B**) Representative BamHI restriction digest showing the band sizes before and after insertion of the selection marker by recombineering. The bands in lane 1 result from a digest of the target plasmid with synthesized insert, and lane 2 contains a digest of the modified plasmid. This plasmid is a mixture of un-targeted and recombined plasmid. The 0.5 and 2.2 kb fragment represents synthesized fragment only and size-shifted cassette containing the selection cassette flanked by homology for insertion into the BAC, respectively. neo: neomycin; *Pgk1*: Phosphoglycerate kinase 1; em7: artificial prokaryotic promoter; BGH pA: bovine growth hormone polyadenylation signal; ori: plasmid origin of replication; Amp: cassette encoding β-lactamase for resistance to ampicillin/carbenicillin. Maps are not drawn to scale.

To generate a selection cassette for dual *E. coli*/ES cell selection, for each gene a fragment with the following components was synthesized and inserted into pUC (Figure 1A): A recognition site for a restriction enzyme (e.g. BamHI), 100 bp 5′ mini homology arm matching the sequence immediately 5′ of the cassette insertion site, a 34 bp FRT site, 71 bp homology to the 5′ end of the mouse *Pgk1* promoter, 60 bp homology to the 3′ end of the bovine growth hormone polyA sequence, a 34 bp FRT site, an 18 bp spacer, a 34 bp loxP site, a 100 bp 3′ mini homology arm matching the sequence immediately 3′ of the cassette insertion site and a recognition site for a restriction enzyme (identical to, or different from, the 5′ site). To prepare the full-length selection cassette, a 1.9 kb EcoRI-BamHI fragment was isolated from PL452 (5) and 10 ng was co-transformed with the modified pUC plasmid into pre-made heat-shocked and recombineering-ready SW102 cells (prepared as described below) using electroporation and the following conditions: 2.5 kV, 25 μF, 200 Ω. After 1 h of outgrowth in 1 ml LB, 100 μl and 200 μl were plated on agar plates containing 50 μg/ml kanamycin and incubated overnight at 32°C. The following day the plasmid was isolated by miniprep (Qiagen). The plasmid prep contains a combination of parent and modified plasmid and the size-shifted targeting cassette was isolated from the backbone using the appropriate enzyme (e.g. BamHI) and gel-purified (GFX kit, GE Healthcare). In most cases three bands are visible on the gel (Figure 1B): the pUC vector backbone, the synthesized and un-modified insert, and the desired 2 kb size-shifted selection cassette resulting from recombination of the *Pgk1*-em7-neo-BGHpA fragment with the cassette homology arms. The cassette was eluted in 10 mM Tris-HCL pH 8.5 and 1 ng was used in the subsequent CoSBR experiment.

**Figure 2.**
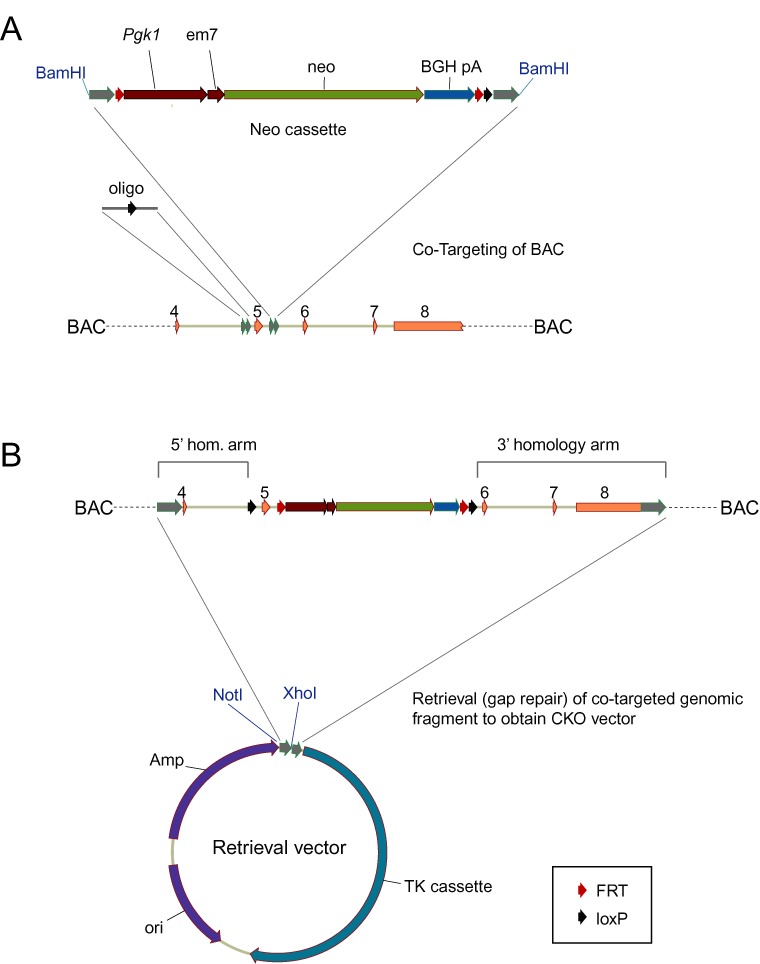
Schematic outline of the CoSBR approach. (**A**) BAC co-targeting by recombineering with a loxP oligo and the neo cassette. Homology arms on the BAC, oligo, neo cassette and retrieval vector are indicated in gray. In this example, the goal is to flank exon 5 by loxP sites to generate a CKO allele. **(B)** After co-targeting, the modified genomic fragment is retrieved by gap repair into an XhoI-linearized retrieval vector, giving rise to the final CKO vector. 5′ and 3′ homology arms for ES cell targeting are indicated. The homology arms are defined as the genomic sequences upstream of the first loxP site and downstream of the 3′ loxP site, respectively. Generally these homology arms are 2.5–3.5 kb in length. After using this vector to generate a targeted allele in mouse ES cells, the selection marker can be removed by Flp expression, leaving a single FRT and two loxP sites in the modified locus. TK: Thymidine Kinase cassette for negative selection on ES cells. Exons are indicated in orange. Other labels as in Figure 1. Maps are not drawn to scale.

The synthesized insert in the pBlightTK retrieval vector has the following configuration: NotI site to enable linearization of the final CKO vector, 200 bp of homology to the very 5′ end of the region to be retrieved from the modified BAC, an XhoI site for linearizing the retrieval vector between the two homology arms, and 200 bp of homology to the very 3′ end of the region to be retrieved (see Figure 2). Five hundred nanogram of the retrieval vector was linearized with XhoI, separated from any un-cut plasmid on a 1% agarose gel and the gel band purified using the GFX kit (GE healthcare). The linearized retrieval vector was eluted in 50 μl of 10 mM Tris-HCL pH 8.5 and 10–30 ng was used for retrieval as described below.

### loxP oligos for CoSBR

A list of all oligos used in this paper is provided in Supplementary Tables S1 and S2. All oligos were obtained from Integrated DNA Technologies, Inc, San Diego, CA, USA. The 200 bp oligos used for CoSBR consist of a 34 bp loxP sequence flanked by two 83 bp homologies matching the sequences flanking the intended insertion site. When included, a total of four phosphorothioate bonds were used, two at the terminal 5′ end and two at the terminal 3′ end. Oligos were received as lyophilized and re-suspended to a final concentration of 5 μM in ddH_2_O. Lagging or leading strand sequences were determined as described above.

### CoSBR

Low salt LB media (LSLB/Lennox) was used throughout: For 1 liter LSLB: 10 g Tryptone, 5 g yeast extract, 5 g NaCl, ddH_2_O to 1 l, autoclave. Pre-mixed Lennox media powder was obtained from Sigma-Aldrich. For the initial experiments, BAC clone integrity was checked by analysis of BAC miniprep DNA using SpeI fingerprinting and comparing to a reference sequence. For routine experiments, to avoid the need for BAC characterization, two BAC clones can be used and pooled into one culture. To introduce pSIM18, a 5 ml overnight culture containing a BAC clone (LSLB containing 12.5 μg/ml chloramphenicol) was prepared for electroporation by cooling on ice and then washing twice in ice-cold ddH_2_O. Supernatant was removed and the pellet re-suspended in a final volume of 100 μl ice-cold ddH_2_O. Ten nanogram of pSIM18 was transformed into 50 μl BAC-containing *E. coli* using electroporation (conditions as above) and after 1 h of outgrowth the culture was diluted 1:50 in 5 ml LSLB containing 12.5 μg/ml chloramphenicol and 100 μg/ml hygromycin and incubated overnight at 32°C. Five hundred microliter of the overnight culture was diluted in 25 ml LSLB containing 12.5 μg/ml chloramphenicol and 100 μg/ml hygromycin in a baffled 50 ml flask, grown to an OD_600_ of 0.55 in a 32°C shaking waterbath (New Brunswick Scientific/Eppendorf), heat-shocked for 15 min in a shaking 42°C degree waterbath, cooled on ice and washed twice in ice-cold ddH_2_O. Supernatant was removed and the pellet re-suspended in a final volume of 200 μl ice-cold ddH_2_O. Fifty microliter heat-shocked and electrocompetent bacteria cells containing the BAC was mixed with 1 ng gel-purified neo cassette and 1 μl loxP oligo (stock concentration 5 μM) and electroporated using a 0.1 cm cuvette and the conditions outlined above. After a 4 h outgrowth at 32°C, the bacteria were plated on LB+25 μg/ml kanamycin plates and the resulting colonies were analyzed by PCR for the presence of the loxP site. Alternatively, for routine CoSBR, 10 μl culture was added to each well of a 96 deep well plate (Corning) containing 500 μl LSLB + 25 μg/ml kanamycin and incubated in a shaking incubator for at least 24 h at 32°C. The plate was screened by PCR using 3 μl as template in a 25 μl PCR reaction with primers flanking the loxP insertion site, using Roche Hi Fidelity polymerase and the following conditions: 1 cycle at 96°C 5 min, followed by 28–35 cycles of 95°C 30 s, 60°C 30 s, 72°C 30 s, then 1 cycle 72°C 2 min. Positive wells were pooled and 500 μl was used to initiate a new culture in 25 ml LSLB containing 25 μg/ml kanamycin, 100 μg/ml hygromycin and 12.5 μg/ml chloramphenicol in a baffled 50 ml flask, grown to an OD_600_ of 0.55. Following the same procedure as above, 50 μl heat-shocked and electrocompetent cells containing the co-targeted BAC was electroporated with 2.5 μl XhoI-linearized and gel-purified retrieval vector (10–30 ng). After 1 h outgrowth at 32°C, the cells were plated on LB + 50 μg/ml carbenicillin plates and incubated overnight at 37°C. pSIM18 has a temperature-sensitive replication origin and the bacteria are cured of pSIM18 by incubation at 37°C. More than 10 carbenicillin-resistant clones were analyzed by sequencing for correct loxP sequence.

### Figures

**Figure 3.**
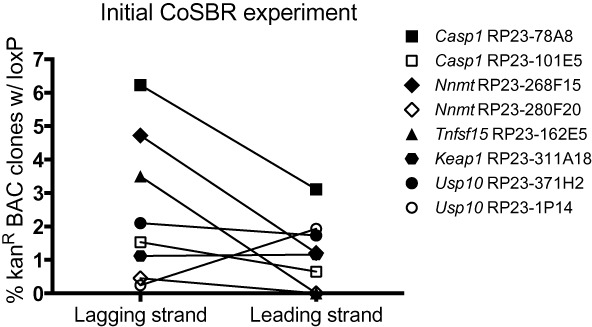
Plot of data from Table 1 (initial CoSBR experiment). Shown are frequencies of kan^R^ BACs containing a loxP site when using either a lagging or leading strand oligo. Results are from analysis of individual colonies obtained from the CoSBR approach outlined in Figure 2. Each experiment (lagging versus leading oligo) was performed once and the related data points are connected by lines for easy comparison. Mouse gene names and BAC clone IDs are indicated.

**Figure 4.**
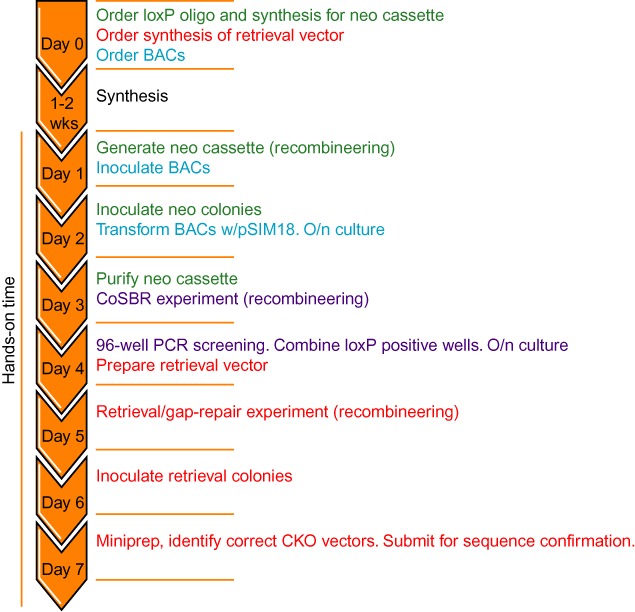
Streamlined 7-day hands-on CoSBR protocol. Most steps are done in liquid bacterial culture, minimizing hands-on time. A detailed protocol is included as supplemental information. Font colors indicate the different steps: neo cassette generation by recombineering (green); preparation of BACs (blue); CoSBR (purple); retrieval (red).

All vector maps (Figures 1 and 2, and Supplementary Figure S1) were created using Vector NTI Advance 11.5.0 (Thermo Fisher Scientific, Inc.), exported as .wmf files and annotated using Adobe Illustrator CS6 (Adobe). Digital agarose gel pictures (.tif format) were imported into Adobe Photoshop CS6 (Adobe), exported in Adobe Illustrator format and subsequently annotated using Adobe Illustrator CS6. Figure 3 was generated in Prism 6 (GraphPad) based on data from Table 1, and annotated in Adobe Illustrator. Figure 4 was generated entirely in Adobe Illustrator.

**Table 1.** Initial CoSBR experiment to determine lagging versus leading strand preference

Gene	BAC clone ID	Gene orientation	Oligo (ID)	# kan^R^ colonies	# loxP sites	Frequency (%)
*Casp1*	RP23–78A8	Sp6 -> T7	Lg (1)	369	23	6.2
			Ld (2)	225	7	3.1
	RP23–101E5	T7 -> Sp6	Lg (2)	131	2	1.5
			Ld (1)	154	1	0.65
*Nnmt*	RP23–268F15	T7 -> Sp6	Lg (3)	318	15	4.7
			Ld (4)	248	3	1.2
	RP23–280F20	T7 -> Sp6	Lg (3)	224	1	0.45
			Ld (4)	148	0	0
*Tnfsf15*	RP23–162E5	T7 -> Sp6	Lg (5)	143	6	3.5
			Ld (6)	178	0	0
*Keap1*	RP23–311A18	T7 -> Sp6	Lg (7)	357	4	1.1
			Ld (8)	345	4	1.2
*Usp10*	RP23–371H2	Sp6 -> T7	Lg (9)	428	9	2.1
			Ld (10)	463	8	1.7
	RP23–1P14	Sp6 -> T7	Lg (9)	422	1	0.24
			Ld (10)	414	8	1.9

Gene orientation: Transcriptional orientation of the gene of interest relative to orientation of genomic insert in pBACe3.6 BAC backbone. Lg: lagging-strand oligo. Ld: leading strand oligo. Oligo (ID) refers to sequences listed in Supplementary Table S1. For each experiment the number of kanamycin-resistant colonies and number of kan^R^ BACs containing a loxP site are listed.

### Plasmids and SW102 cells

All recombineering reagents described in this study (SW102 cells plus PL452 and pSIM18 plasmids) were obtained from NCI (http://ncifrederick.cancer.gov/research/brb/recombineeringInformation.aspx.) pBlightTK (sequence and/or DNA) is available from us upon request.

## RESULTS

### Generation of the components for CKO vector construction using a combination of gene synthesis and recombineering

Current methods for generating the starting material needed for producing a CKO vector using recombineering (targeting cassettes and retrieval vector) rely on either PCR, molecular cloning, or a combination of both. To ensure error-free construction of gene targeting vectors, these PCR products will have to be subcloned and fully sequenced prior to use, adding end-user hands-on time.

Custom DNA synthesis has in recent years become a reliable and cost-efficient alternative to end-user PCR and molecular cloning of cDNAs in cases where a high quality reference sequence exists. Accordingly, the high quality of the genomic sequence of the inbred C57BL/6J mouse genome assemblies (e.g. NCBI37/mm9 build) makes direct DNA synthesis of mouse genomic DNA sequences an attractive alternative to PCR amplification from a BAC or genomic DNA source. Although the synthesis cost per DNA base pair is rapidly decreasing, due to increased turnaround time and difficulties with synthesis of low complexity, repetitive and GC-rich regions, custom DNA synthesis is not yet a viable cost-effective solution to full-length (6–10 kb) gene targeting vectors. Instead, the power of DNA synthesis can be realized when combined with other technologies such as recombineering. We routinely use a simple and very efficient PCR- and cloning-free method for the rapid generation of error-free intermediate components for CKO vectors. In this approach, DNA synthesis is used to produce only the ‘critical regions’ (mini homology arms, loxP and FRT sites), and these pieces are then used directly for recombineering. To produce a cassette for inserting FRT-neo-FRT-loxP, the critical regions (loxP and FRT sites and homologies to the gene of interest) are synthesized, and then a full-length neo selection marker is inserted into this sequence by recombineering (Figure 1A). The synthesized sequence is flanked by restriction sites so the final cassette can be released from the vector. By only synthesizing the critical regions and not the full-length selection markers, synthesis turnaround time is fast and synthesis cost is low. After receiving the targeting cassette plasmid containing the newly synthesized sequences, a selection marker is simply inserted by co-transforming the plasmid along with the corresponding linear selection marker (isolated from plasmid PL452) into pre-made heat-shocked and electrocompetent recombineering-proficient SW102 cells (19). The size-shifted fragment that now contains the selection marker flanked by synthesized sequence can then be isolated and purified (Figure 1B) and the cassette used directly for BAC recombineering. To produce the retrieval vector, a short sequence consisting of a unique linearization restriction site (e.g. NotI), a short (200 bp) homology arm corresponding to the extreme 5′ end of the region to be retrieved from a BAC clone, another unique restriction site (e.g. XhoI) and another short homology arm corresponding to the extreme 3′ end of the region to be retrieved, is assembled. This sequence is custom ordered as one synthesized DNA fragment cloned directly into our preferred gene targeting vector backbone (pBlightTK (20)), which contains the negative ES cell selection marker Herpes Simplex Virus Thymidine Kinase. While traditional methods (PCR, cloning) can be used instead of gene synthesis to generate the components needed for recombineering and CoSBR, we find that gene synthesis is a very convenient and cost-effective alternative, significantly reducing hands-on time and eliminating the need for end-user sequence verification of the components.

### Lagging-strand oligo is generally preferred over leading-strand oligo in CoSBR

A method for co-selection targeting of the bacterial genome (CoS-MAGE) has been described (12,13) and we hypothesized that this principle could be applied to BAC recombineering as well. BACs differ from the bacterial genome in two important ways: First, BACs are replicated unidirectionally from OriS whereas the bacterial genome is replicated bi-directionally from OriC. Second, BACs are usually in the 100–200 kb size range whereas the *E. coli* genome is ∼4.6 Mb (25–50 times larger than the average BAC) so replication of a BAC is faster than *E. coli* replication, potentially providing a shorter time window for BAC recombineering. We hypothesized that co-transformation of a kanamycin selectable marker (FRT-*Pgk1*-em7-neo-FRT-loxP) along with a single-stranded oligo containing a loxP site flanked by short homologies, followed by selection for kanamycin-resistant BACs, would result in targeted BACs where a fraction would contain the loxP site in addition to the positively selected neo cassette (Figure 2A). This approach would obviate the need for any further modification of the genomic region, and the modified genomic fragment could then be retrieved from the BAC into a plasmid backbone (retrieval vector), resulting in the final CKO targeting vector (Figure 2B). According to the current model for recombineering using single-stranded DNA oligos (8,9), an oligo can serve to prime Okazaki fragment synthesis during replication, and thus lagging-strand oligos should be more efficient than leading-strand oligos for recombineering (9). To test this hypothesis, we evaluated whether the bias toward lagging versus leading-strand oligos would also be true in a co-selection scheme where the directly selectable marker is double-stranded (the neo cassette) and the co-selected loxP oligo is single-stranded. We obtained one or two BAC clones for five different genes (Table 1) and designed the corresponding lagging and leading-strand loxP-containing oligos as described in Materials and Methods (Supplementary Table S1). We also generated gene-specific FRT-*Pgk1*-em7-neo-FRT-loxP cassettes as described above. To eliminate bias due to rearranged and incorrect BACs, we first characterized each BAC by restriction enzyme fingerprinting as described previously (19) (data not shown). We then performed CoSBR for all BACs individually, as outlined in Figure 2, by co-transformation of the linear selection cassette along with loxP oligo. As it has been shown that addition of phosphorothioate bonds can prevent degradation of single-stranded oligos by exonucleases and thereby possibly increase recombineering efficiency (8,9,21), two phosphorothioate bonds at both the 5′ and 3′ end of the oligos were added (Supplementary Table S1). The optimal stock concentration of oligo (5 μM) for CoSBR was determined empirically (data not shown) using a fixed amount of selection marker (1 ng) that we have found results in, on average, 200–400 kan^R^ colonies. Increasing oligo concentration above 5 μM resulted in a significant loss of viable bacteria after electroporation. After co-transformation and outgrowth to allow for the targeted neo/kan resistance marker to be expressed, we plated all the culture on agar plates with 25 μg/ml kanamycin. All resulting colonies were picked and grown overnight in 96-well culture plates in the presence of kanamycin and the next day analyzed by PCR to determine frequencies of kanamycin-resistant (targeted) BACs that also contain the loxP site. In total, we targeted five genes using eight BACs along with oligos targeting both strands, in order to evaluate the efficiency of CoSBR. The results of the initial CoSBR experiment are summarized in Table 1 and depicted in Figure 3. Importantly, our data shows that co-targeting of a BAC with a double-stranded selection marker and a single-stranded oligo is indeed possible. As expected, lagging-strand oligos were preferred over leading strand oligos in most cases. In one case there was no obvious preference of lagging versus leading strand (*Keap1*, RP23–311A18) and in one case the leading strand was preferred over the lagging-strand oligo (*Usp10*, RP23–1P14). Importantly, while we did observe experiments in which the leading-strand oligo failed to generate any clones with a loxP site, loxP-targeted BACs were obtained from the lagging-strand oligo in all of these experiments. To exclude the possibility of incorrect BAC clone annotation in the *Usp10*, RP23–1P14 experiment where the leading strand oligo was strongly favored, we confirmed that the orientation of the insert was indeed as annotated using the UCSC genome browser (data not shown). The observed preference for leading-strand over lagging-strand in the *Usp10*, RP23–1P14 experiment was not gene-dependent since the data using a separate BAC for *Usp10* suggested either a slight preference for lagging strand or no preference at all (Figure 3). While our initial data was based on single, and not repeated, experiments, the fact that we obtained co-targeted BACs for all five genes tested suggested that the CoSBR approach should be generally applicable.

The distances between the insertion site of the loxP and the insertion site of the selection marker were gene-specific and varied between 500 bp and 2.1 kb. We observed no correlation between loxP-neo distance and efficiency of CoSBR (data not shown).

In conclusion, co-targeting of BACs using the CoSBR approach is feasible and our data is in agreement with the current model for recombineering and a preference for the lagging-strand oligo, even when co-transformed with a double-stranded selection marker.

### An efficient 96-well format CoSBR protocol for rapid production of CKO vectors

Encouraged by the initial efficiency of our CoSBR approach to BAC modification, we sought to further develop the approach by keeping most of the steps in liquid bacterial culture to avoid the need for plating and picking individual colonies until the final step (after retrieval or ‘gap repair’). In our initial experiments (Table 1), the number of kan^R^ colonies obtained with 1 ng selection cassette varied between 131 and 463 colonies. If the liquid culture (after electroporation and outgrowth) is distributed into a single 96-well culture plate instead of plating the culture on agar plates, we would thus expect ∼1–4 kan^R^ colonies seeding each well. After an overnight culturing period with kanamycin selection we would expect growth in all wells and we would expect most wells to be ‘polyclonal’ in origin, i.e. the kan^R^ bacteria are derived from more than one individually targeted BAC-containing bacterium. Since only kan^R^ bacteria can grow in the selective media, we expect that a given well contains either bacteria (mono- or polyclonally derived) that harbor targeted kan^R^ BACs without a loxP site (‘neo only’) or it contains a mixture of ‘neo-only’ bacteria and bacteria harboring a co-targeted BAC (‘CKO’). Some wells might exclusively contain CKO bacteria, although we would expect that most wells will also contain neo-only bacteria based on the co-targeting frequencies observed in the initial experiment (0.24–6.2%).

Importantly, a PCR screen to distinguish CKO-positive wells from neo-only wells cannot distinguish ‘neo-only’ from *residual* (un-targeted) BACs. It is therefore important to allow sufficient culture time for kan^R^ bacteria to become the dominant population in each well. In other words, PCR screening should be done after the 96-well o/n cultures reach a density where the majority of bacteria contain the modified BAC. It will then be possible to identify which of the wells harbor bacteria containing loxP positive BACs. In practical terms, we find that growing the 96-well culture plate for at least 24 h provide enough time for the kan^R^ bacteria to become the dominant population and at that time the residual un-selected bacteria only contribute minimally as template for the PCR.

**Figure 5.**
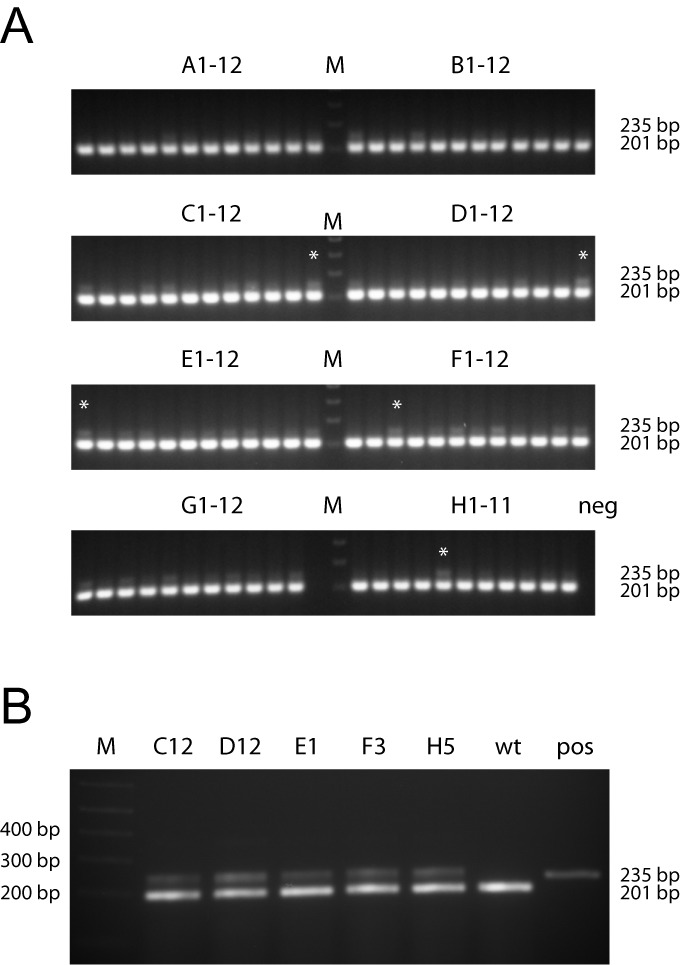
Representative analysis of a 96-well CoSBR experiment. (**A**) 1.5% agarose gel electrophoresis of PCR products generated using primers flanking the 5′ loxP insertion site in a *Cdh11* BAC (RP23–60C23). Wild-type amplicon is 201 bp, and the loxP containing amplicon is 235 bp. White asterisks denote five wells chosen for subsequent retrieval. (**B**) Repeated PCR analysis of the five chosen wells from (A) to verify presence of loxP site. G12: this well had no bacterial growth. H12: no inoculation (neg: negative control). wt: wild-type, un-modified BAC. pos: plasmid positive control with loxP site (final CKO vector).

Based on the assumption of 1–4 kan^R^ founder bacteria per well and data from our initial experiments, we developed and tested a more streamlined CoSBR protocol for generation of CKO vectors (outlined in Figure 4). Our 96-well PCR screening strategy uses a small amplicon with primers located outside of the loxP oligo homology arms so that the increase in amplicon size resulting from the extra 34 bp from the loxP site can be easily used to distinguish the two types of kan^R^ BACs. After obtaining the synthesized fragments (starting plasmid for the targeting cassette, loxP oligo and retrieval vector), the CoSBR protocol can be completed in 7 days or less with minimal hands-on time. We applied CoSBR to generate two additional CKO vectors using 2–3 BACs per gene along with the corresponding lagging-strand oligo. The data for both genes is summarized in Table 2, and the result from screening a 96-well plate from one of these projects is shown in Figure 5A (*Cdh11*, RP23–60C23). The oligos used for CoSBR are 200 bp and a fraction of these oligos will most likely contain sequence errors. To make sure we are generating a CKO vector completely without any errors either in the loxP sequence or in the sequence flanking the loxP site and introduced by the oligo, for each experiment we combined five loxP-positive wells for subsequent retrieval/gap-repair. To verify that all five wells picked indeed contain bacteria with the loxP-modified BAC, the PCR was repeated (Figure 5B). By combining bacteria from five individual wells, the modified genomic fragment is retrieved from several independently targeted BACs, increasing the likelihood of identifying an error-free vector to be used for ES cell targeting. 36% of the wells from the RP23–60C23 experiment (Figure 5) were loxP positive (Table 2). After retrieval, 6/21 plasmids (29%) contained the loxP site and the remaining plasmids contained the neo cassette only. This suggested an average loxP:neo-only ratio of 1:2 in the five wells combined for retrieval. Of these six loxP positive plasmids, all contained an error-free loxP site. In the other *Cdh11* experiment 2/95 wells were positive. Since identical neo cassettes were used in these two experiments and the only difference is the orientation of the oligo and the BAC genomic insert, it is possible that for this gene one orientation is favored over the other. Retrieval was not done from the second experiment. Data for the other gene, *S100A8*, is also summarized in Table 2. In one experiment 60/95 (63%) of the wells were loxP-positive, 6/17 (35%) of the retrievals contained the loxP site and 5/6 had an error-free loxP sequence. For the other S100A8 experiment two BACs with the same back-bone orientation were combined and 15/95 (54%) of the wells were loxP positive. Retrieval was not done from the second experiment.

**Table 2.** Summary of results from two additional CKO projects using the optimized liquid culture CoSBR protocol

Gene	BAC clone ID	Kan^R^ wells w/loxP	Retrievals w/lox	Retrievals with error-free loxP
*Cdh11*	RP23–60C23	34/95 (36%)	6/21 (29%)	6/6 (100%)
	RP23–35E14	2/95 (2%)	n.d.	n.d.
*S100A8*	RP23–6E18	60/95 (63%)	6/17 (35%)	5/6 (83%)
	RP23–189H20/229K18	51/95 (54%)	n.d.	n.d.

One or more BAC clones were used per project along with a matching lagging-strand oligo. Experiments were done in 96-well plates. Retrieval minipreps were analyzed for presence of loxP and loxP-positive CKO vectors were subsequently sequenced. n.d., not done.

In summary, we have developed an efficient co-targeting strategy for efficient BAC modification and we have shown that this method can be effectively applied to the construction of conditional KO gene targeting vectors.

## DISCUSSION

In this study we describe CoSBR, a highly efficient approach for the construction of conditional KO targeting vectors. CoSBR improves upon existing methods for (selection-free) oligo-mediated recombineering of BACs (22,23) by eliminating the need for a significant amount of screening effort and/or two rounds of BAC targeting. Gene targeting vectors for ES cells require a positive selection marker such as neo, and in our CoSBR approach we take advantage of a dual bacterial/eukaryotic selection marker to enrich for bacteria that contain BACs which have undergone recombineering. We show that a significant fraction of kan^R^ bacteria contain a BAC clone that is co-targeted by the oligo. For applications other than the generation of gene targeting vectors where it is not desirable to leave a selection marker in the BAC, we speculate that it should be possible to modify any location of a BAC clone by CoSBR: By simply swapping the selection marker in the BAC backbone for another marker one could enrich for BAC clones that have undergone recombineering and then screen these for the non-selectable event (e.g. introduction of a point mutation). By virtue of the differences in gene structure, the conditional KO vector designs used in the present study vary in the loxP-to-neo distance. We did not observe a correlation between proximity of loxP insertion site and neo insertion site, but it is presently not known if efficiency of CoSBR decreases significantly when the distance between selectable marker and non-selected event is increased beyond a few Kb. However, it has been shown that CoS-MAGE is efficient over long distances in the *E. coli* genome (12) and it is thus possible that one could modify a BAC genomic insert even if >100 kb away from the backbone and selection marker. While beyond the scope of this study, it will be interesting to test this approach to BAC recombineering in future studies and to define the efficiency range of the CoSBR approach.

According to the established model for recombineering (8,9), when introduced into bacteria, a double-stranded cassette is first converted to single-stranded DNA after which it can serve to prime Okazaki fragment synthesis. Thus, the preference for lagging strand should apply to both oligos and double-stranded DNA cassettes for BAC recombineering. In our study we used a combination of a single-stranded DNA oligo, protected by phosphorothioate bonds and a double-stranded cassette isolated from a plasmid. Our data supports the preference for lagging strand-matching oligos for recombineering in agreement with the current model, whereas we cannot conclude from our data if there is a preference for the lagging-strand-matching half of the double-stranded DNA cassette.

In this study all the BAC clones were characterized by restriction enzyme ‘fingerprinting’ prior to use, to avoid any bias due to the integrity of the BAC clone. In our experience, the most likely explanation for a non-successful outcome of a BAC recombineering experiment is that the BAC clone is either incorrectly annotated or the clone has undergone rearrangements so the sequence of interest is absent in the BAC. In our hands, ∼10% of the RP23 library BACs are incorrect. However, characterizing BAC clones can be tedious, especially in a high-throughput setting, and we find that by combining two unique BAC clones with the same BAC insert polarity for each project, the risk of working with an incorrect BAC is significantly reduced as at least one of the two BACs is usually correct (the risk of both BACs being incorrect is ∼1%).

The approach described here to generate the components for CoSBR successfully combines the advantages of recombineering with the end-user convenience of custom DNA synthesis. By having the key components needed for recombineering synthesized rather than generated by PCR followed by cloning and sequence verification of these components, the hands-on time for generating gene targeting vectors is significantly reduced since only the actual recombineering steps remain. We find that the DNA synthesis turn-around time is approaching the time it would take to obtain a BAC template for PCR, generate the PCR products, clone and sequence-verify the components. Since all the components (the synthesized pieces and the selection markers) have been sequence-verified prior to initiating the CoSBR experiment, one only needs to check the sequence of the loxP site, and when verifying the sequence of the final targeting vectors we therefore find a 100% concordance between expected and actual sequence. In our optimized CoSBR protocol, most of the steps are performed in liquid bacterial culture, and our approach is therefore particularly useful when simultaneously generating multiple vectors. CoSBR should therefore be easy to scale up for high-throughput generation of gene targeting vectors.

Realistic alternatives to modification of the mouse genome via gene targeting in mouse ES cells now exist. In particular, CRISPR/Cas9 technology promises to revolutionize the field of genome engineering. CRISPR/Cas9 is a very efficient approach to the generation of conventional KO alleles and for introducing small point mutations in the mouse genome (24). For more complex genome engineering including conditional KO (25) and conditional knock-in alleles, as well as knock-in of large pieces of DNA and for humanization of mouse genes, CRISPR/Cas9 technology has yet to be widely adopted by the community. Furthermore, for situations where there is no flexibility in the engineering design and where an efficient sgRNA is not available, ES cells continue to provide an efficient means of engineering the mouse genome. As such, there is still a need for new and improved approaches to the generation of gene targeting vectors, and we have shown here that CoSBR is an efficient method for BAC engineering and in particular for the generation of CKO vectors.

## SUPPLEMENTARY DATA

Supplementary Data are available at NAR Online.

SUPPLEMENTARY DATA
