# Supplementary material for: Efficient conditional knockout targeting vector construction using co-selection BAC recombineering (CoSBR)
Source: Nucleic Acids Res. 2015 Jun 18;43(19):e124. doi: 10.1093/nar/gkv600 (PMC4627060; doi:10.1093/nar/gkv600)
Supplement: SUPPLEMENTARY DATA [file supp_43_19_e124__index.html]

Efficient conditional knockout targeting vector construction using co-selection BAC recombineering (CoSBR) — SUPPLEMENTARY DATA 

# Efficient conditional knockout targeting vector construction using co-selection BAC recombineering (CoSBR)

## SUPPLEMENTARY DATA

- SUPPLEMENTARY DATA
