# Supplementary material for: Efficient conditional knockout targeting vector construction using co-selection BAC recombineering (CoSBR)
Source: Nucleic Acids Res. 2015 Jun 18;43(19):e124. doi: 10.1093/nar/gkv600 (PMC4627060; doi:10.1093/nar/gkv600)
Supplement: SUPPLEMENTARY DATA [file supp_gkv600_nar-01247-met-h-2015-File007.pdf]

Figure S1

A

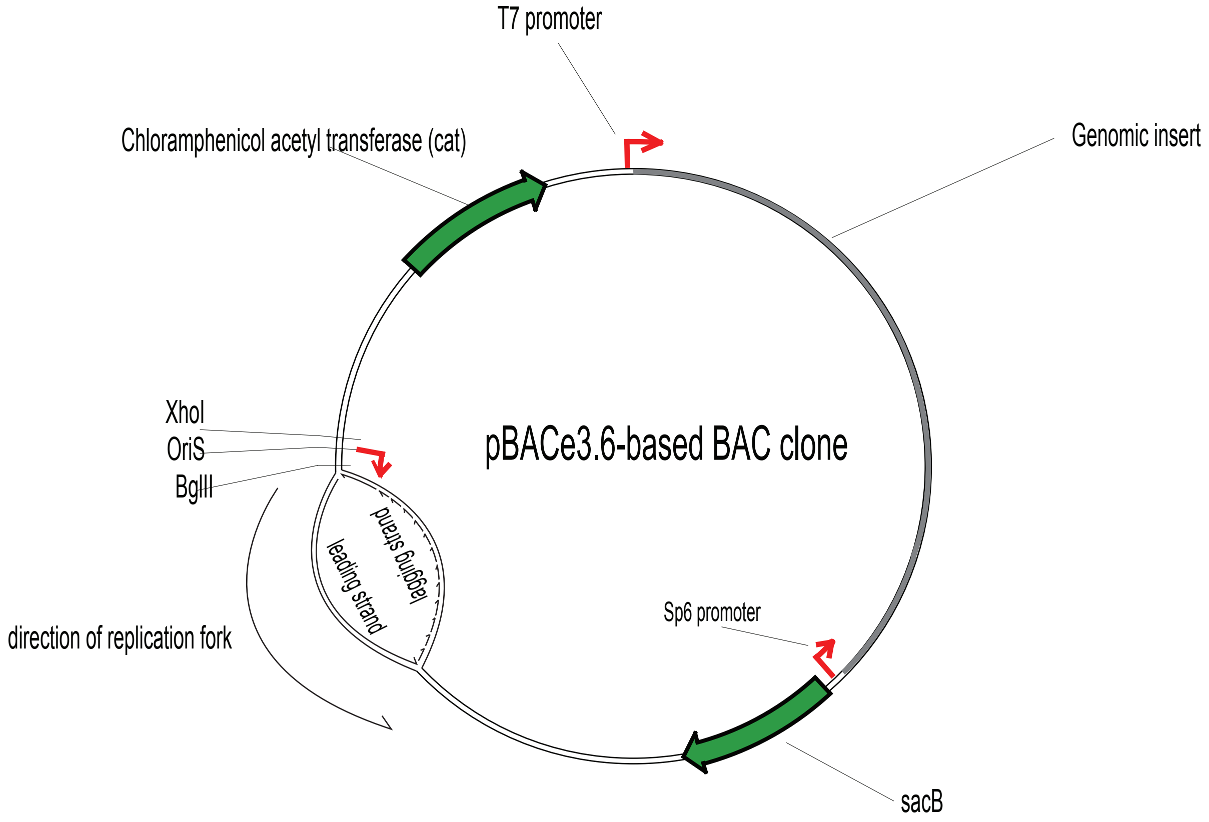

B

UCSC Genome Browser on Mouse July 2007 (NCBI37/mm9) Assembly
Chr9

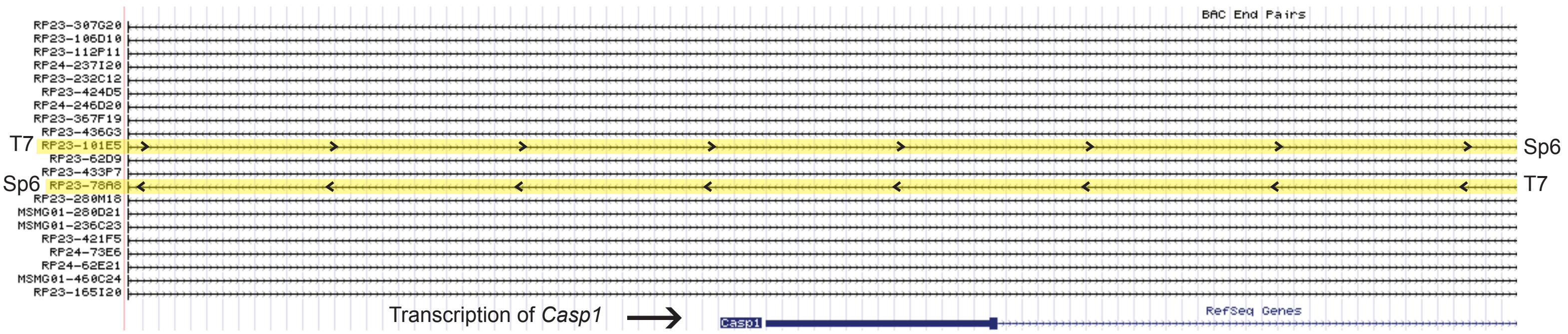

Figure S1. Schematic overview of BAC replication in pBACe3.6-based BAC clones, and determination of lagging and leading strands. (**A**) BAC replication is initiated at OriS and the replication fork proceeds in the direction indicated. With respect to the genomic insert, the replication fork reaches the Sp6 promoter first, proceeds through the genomic insert, then T7, and finally the remaining part of the BAC vector backbone is replicated. (**B**) The two *Casp1* BACs used in this study (RP23-101E5 and RP23-78A8) are highlighted, and the orientation of the genomic insert with respect to Sp6/T7 is indicated. Transcription of *Casp1* is also indicated. The lagging strand has the same 5'-3' polarity as the T7 coding strand. Thus, the lagging strand polarity of the RP23-101E5 clone is identical to the *Casp1* coding strand polarity and homology arms for the loxP oligo for this clone should match the coding strand sequence (see oligo #2). Polarity is opposite in the RP23-78A8 clone and homology arms for the loxP oligo for this clone should therefore match the *Casp1* non-coding strand sequence (see oligo #1).

Table S1. CoSBR oligos. Asterisks (*) denote phosphorothioate bonds. loxP sequence is indicated in bold. All oligos listed 5' to 3'.

Oligo	Gene	Sequence
1	*Casp1*	T*A*AGAGGTTTTAATTTATGACCACAAGGTGTTACTTCAGATACAGAGTGGACCAAGGAATG<br>GTTGTTTTAGAAGGCTAATAC**ATAACTTCGTATAGCATACATTATACGAAGTTAT**TAATCTAA<br>GATGTAGTAGTTACCGTCTGCACCTACAAAATTCCAAACTTTCTACAGTACAGCAATTCCAAT<br>TGTCTGTCCAT*C*T
2	*Casp1*	A*G*ATGGACAGACAATTGGAATTGCTGTACTGTAGAAAGTTTGGAATTTTGTAGGTGCAGAC<br>GGTAACTACTACATCTTAGATTA**ATAACTTCGTATAATGTATGCTATACGAAGTTAT**GTATTA<br>GCCTTCTAAAACAACCATTCCTTGGTCCACTCTGTATCTGAAGTAACACCTTGTGGTCATAAA<br>TTAAAACCTCT*T*A
3	*Nnmt*	G*CATAGGTATTTGTGAGATGCCTGACTTGTTACACAGGTTCTGGGACCTGAATGCTGGCCCT<br>CATCTCTGGACCATCACTCCA**ATAACTTCGTATAATGTATGCTATACGAAGTTAT**GGCCTTAA<br>AATCAGTTGTTACACAACAACAACGACAACACCACCACCACCACAACAAAAATGGCACAATCC<br>CAGTAAAGAC*A*C
4	*Nnmt*	G*T*GTCTTTACTGGGATTGTGCCATTTTTGTTGTGGTGGTGGTGGTGTTGTCGTTGTTGTTG<br>TGTAACAACTGATTTTAAGGCC**ATAACTTCGTATAGCATACATTATACGAAGTTAT**TGGAGTG<br>ATGGTCCAGAGATGAGGGCCAGCATTCAGGTCCCAGAACCTGTGTAACAAGTCAGGCATCTCA<br>CAAATACCTAT*G*C
5	*Tnfsf15*	A*A*AGACAGGAGAAAGGAAGGCCATGGGGGTCTCTAGAAAAGGTGTGCAGAGCCGAGAGGCA<br>AGATTCCCACCAACTGAGATAC**ATAACTTCGTATAATGTATGCTATACGAAGTTAT**CCCAATG<br>CTGTTCTCTCCCAGCGAGGACCTAGGCTCAGACAGCAGCAGAGTCTTATGGGCAGCCGTGTGG<br>GTTTTTGAGTT*A*A
6	*Tnfsf15*	T*T*AACTCAAAAACCCACACGGCTGCCCATAAGACTCTGCTGCTGTCTGAGCCTAGGTCCTC<br>GCTGGGAGAGAACAGCATTGGG**ATAACTTCGTATAGCATACATTATACGAAGTTAT**GTATCTC<br>AGTTGGTGGGAATCTTGCCTCTCGGCTCTGCACACCTTTTCTAGAGACCCCCATGGCCTTCCT<br>TTCTCCTGTCT*T*T
7	*Keap1*	A*T*TAGTCCAATGCTGGGATTAAAGGCCAGACTAGGTTCAATTGTTTATTTTGATATAAGAT<br>CTTACTATGTTGTCCTAGCTGG**ATAACTTCGTATAATGTATGCTATACGAAGTTATT**CTGGAA<br>CTGGCTTATGGGGACCAATCTGGCCTTGAATTCAGACATCCTCTTGCCTCTGCCTCCCCTGAT<br>ATGGGGGATTA*T*A
8	*Keap1*	T*A*TAATCCCCCATATCAGGGGAGGCAGAGGCAAGAGGATGTCTGAATTCAAGGCCAGATTG<br>GTCCCCATAAGCCAGTTCCAGA**ATAACTTCGTATAGCATACATTATACGAAGTTAT**CCAGCTA<br>GGACAACATAGTAAGATCTTATATCAAAATAAACAATTGAACCTAGTCTGGCCTTTAATCCCA<br>GCATTGGACTA*A*T
9	*Usp10*	A*C*ACAGATTCGCCCCAATCTGAACCAAACAGTGTAGGAATGCTTTCAATCGTTTACCACTG<br>CTAAATACACTGCAACGGAGAC**ATAACTTCGTATAGCATACATTATACGAAGTTATT**GGGATA<br>CTGCCTGAGTGTCAGGCAGGCAGGATGATCCCAACCCCAACACTCCATCTGTTAACAGTGGGA<br>ACTGAAGAGTC*A*G
10	*Usp10*	C*T*GACTCTTCAGTTCCCACTGTTAACAGATGGAGTGTTGGGGTTGGGATCATCCTGCCTGC<br>CTGACACTCAGGCAGTATCCCA**ATAACTTCGTATAATGTATGCTATACGAAGTTAT**GTCTCCG<br>TTGCAGTGTATTTAGCAGTGGTAAACGATTGAAAGCATTCCTACACTGTTTGGTTCAGATTGG<br>GGCGAATCTGT*G*T
11	*Cdh11*	A*C*CTCAGAATTAAACATTTGCAAATCCATCAGCCTCATCATTACCTTGGGTCATAGCAGTT<br>ATCTAAGACTCCATTTCTCTAT**ATAACTTCGTATAATGTATGCTATACGAAGTTATT**CAGAAA<br>ATGATGATGGAAATCACTTCGTCTCATGATATGTGTGTGACTGTATGTATGTGCACACATGTG<br>TACATAGTGTA*A*C
12	*Cdh11*	G*T*TACACTATGTACACATGTGTGCACATACATACAGTCACACACATATCATGAGACGAAGT<br>GATTTCCATCATCATTTTCTGA**ATAACTTCGTATAGCATACATTATACGAAGTTAT**ATAGAGA<br>AATGGAGTCTTAGATAACTGCTATGACCCAAGGTAATGATGAGGCTGATGGATTTGCAAATGT<br>TTAATTCTGAG*G*T
13	*S100A8*	A*A*GGAAGTGGAATGGCGTAGAGCCTTCTAGCAGTGTCTAGCAGAAGAGGGCAGGAAGTGTT<br>TAGTGTGGAGAATTTGCTACAC**ATAACTTCGTATAATGTATGCTATACGAAGTTAT**TTGTGTG<br>TGTGTGTACTAAAGTCTCTCCAATGCTCTCTCCTACCCCTTCCCATCAGAGATAGAGCCATTT<br>TCTGGGTTTAG*T*T
14	*S100A8*	A*A*CTAAACCCAGAAAATGGCTCTATCTCTGATGGGAAGGGGTAGGAGAGAGCATTGGAGAG<br>ACTTTAGTACACACACACACAA**ATAACTTCGTATAGCATACATTATACGAAGTTAT**GTGTAGC<br>AAATTCTCCACACTAAACACTTCCTGCCCTCTTCTGCTAGACACTGCTAGAAGGCTCTACGCC<br>ATTCCACTTCC*T*T

Table S2. PCR primers for detection of loxP-containing BACs. F: forward primer. R: reverse primer. All oligos listed 5’ to 3’.

Oligo	Gene	Sequence
15	*Casp1 F*	GTCTGGATGTGAACAGAATTCTCCTGG
16	*Casp1 R*	CCACAAGGTGTTACTTCAGATACAGAGTGG
17	*Nnmt F*	AGAGCCTCTCCTCTCTGCTCC
18	*Nnmt R*	TCAAAAGGGAACAGGCAGAGC
19	*Tnfsf15 F*	AATCACCTCAGATACAGCAGG
20	*Tnfsf15 R*	CCATAAGACTCTGCTGCTGTC
21	*Keap1 F*	GACATCTTCCTCTCCCTTCCAAGTG
22	*Keap1 R*	GGTCCCCATAAGCCAGTTCCAG
23	*Usp10 F*	TGGCTCACTTCTGCCAGTGGTG
24	*Usp10 R*	GCAACTGGCCATGACATCACGT
25	*Cdh11 F*	GAATAAGAGAGGAAGAACATGGACCTCAG
26	*Cdh11 R*	CACATAGGTTGAAAGTTACACTATGTACACATGTG
27	*S100A8 F*	CAGCACCGATCAAATGCTTGAGG
28	*S100A8 R*	CTTTGACCTGGCCTGTACCACTCAG

CoSBR protocol
Conditional Knock-Out (CKO) vector construction

Robert J. Newman, Merone Roose-Girma and Søren Warming

The protocol basically involves 4 steps, color-coded for easy overview: BAC Preparation, Cassette Preparation, Co-targeting, and Retrieval.

The day numbering below should be considered *experimental* days 1, 2, 3, and so on. It is possible to stop the procedure at several steps if necessary. However, as the day numbers indicate, it is possible to go from start to finish in 1 week (after BACs and oligos have arrived), or even faster when you become familiar with the procedure. To save more time, at several steps you can inoculate colonies in the morning in a small volume of media, and after 6 hours isolate miniprep DNA, thereby speeding up the process. Retrieval vector and targeting cassette can be prepared using alternatives to gene synthesis (such as PCR). This might speed up the process, but the retrieval plasmid and cassette sequence will each have to be sequence-verified prior to the CoSBR experiment.

Day 0

BAC Preparation

- Order two BACs covering your genomic region by using a genome browser (http://genome.ucsc.edu/), making sure it only maps once in the mouse genome. If you plan to use C57BL/6 ES cells, use BACs from the RP23 or RP24 libraries. Ensure that the genomic fragments were cloned into the BAC vector backbone in the same direction so the lagging strand oligo matches both BACs. Orientation is indicated by arrows on the 'BAC end sequence' track on the UCSC genome browser.

Cassette preparation

- Order synthesis of retrieval vector, with two 200 bp retrieval arms (200 bp 5' homology arm and 200 bp 3' homology arm with XhoI, or another unique restriction site, in between) cloned into pBlight-TK (or your favorite retrieval vector backbone).
- Order mini-targeting cassette for inserting the FRT/loxP flanked selection marker into your construct. Use the template below to make sure no unwanted mutations are introduced and that loxP and FRT sites are 100% correct.
- Order 200 bp loxP oligo. The loxP should be flanked by 83 bp homology arms. Ensure that when targeted, it is in the same orientation as the loxP on your selection cassette. The oligo should be ordered so that it acts as a primer for Okazaki fragment synthesis. Replication of the BAC is in the opposite direction of the arrows on the 'BAC end sequence track' on the

UCSC genome browser, and the lagging strand therefore has the same direction as the arrows. If the gene of interest is transcribed in the same direction as the T7 promoter in the BAC, then the lagging strand is equal to the coding strand of the gene. If the gene of interest in transcribed in the opposite direction as the T7 promoter, then the lagging strand is equal to the non-coding (template) strand.

- You may choose to add phosphorothioate modified bases as the two terminal 5' and 3' bases.
- Digest PL452 with EcoRI and BamHI and gel-purify 1.9 kb fragment. Elute in 10 mM Tris-HCL pH 8.5.

Day 1 (Your BACs, oligo, and synthesized vectors have now arrived)

BAC preparation

- The BACs are in the DH10B bacterial strain. Keep the BACs @-80° or on dry ice.
- From the glycerol stock, use a sterile toothpick to initiate an overnight (o/n) culture for each BAC. 5 ml LSLB w/ 12.5 µg/ml chloramphenicol (15 ml Falcon tube). Use a 32°C incubator.

Prepare cassette for co-targeting experiment

- Co-transform 10 ng 1.9 kb EcoRI-BamHI fragment isolated from PL452 with 100 ng synthesized circular cassette plasmid into heat-shocked and electrocompetent SW102 cells *(see* Box 2 *below for making pre-made SW102 cells)*
- Incubate @ 32°C for 1 hour
- Plate on kanamycin plates (50 µg/ml)
- Incubate o/n @ 32°C

Prepare retrieval vector

- Linearize 500 ng of the pBlightTK+homology retrieval vector with XhoI (introduced unique restriction site) for 1-2 hours
- Run the digest either several hours at 50-70 V, or o/n at low voltage. *It is very important not to digest too much plasmid. This is to avoid carry-over of any uncut plasmid. It is important to have perfectly linearized plasmid for successful retrieval.*
- Cut out gel band (if your gel runs o/n, cut out gel bands next morning)

Day 2

BAC preparation

- Put the culture(s) in an ice-slurry for 5 min. to cool. Also cool 50 ml ddH_2O on ice.
- Spin down the culture(s) @ 2,800 x g for 3 min. in a pre-cooled centrifuge @ 0°C.

- Pour off ALL the supernatant, gently tap on a paper towel to remove the residual liquid, and place the tube in the ice bucket.
- Add 1 ml ice-cold ddH_2O and, keeping it on ice, gently swirl the tube to get the pellet resuspended.
- When the cells are completely and evenly resuspended, add 9 ml ice-cold ddH_2O, close the cap, and invert a couple of times, then spin again for 3-5 min.
- Repeat the washing step and spin one last time.
- After the final spin, the pellet is very loose, so be careful when removing the supernatant the last time. *You should wait at the centrifuge to the last spin is completed, or you might not get there in time to avoid losing the pellet... Try to pour away all the supernatant while keeping an eye on the pellet while it starts sliding down the side of the tube.*

Box 1

- Resuspend the pellet in the small remaining volume (around 50-75 µl) by swirling. *If too much volume, then transfer to an eppendorf tube, briefly spin the tube at full speed for 15 sec. remove excess water, and resuspend pellet gently in remaining volume.*
- In an eppendorf tube (on ice), mix 1 µl (10 ng) pSIM18 plasmid (contains all the recombineering genes) with 25 µl freshly prepared electrocompetent cells for each of the two BACs. Transfer to a 0.1 cm cuvette and electroporate. Use preset standard conditions for E. coli (1.8 kV, 25 µF, 200 Ω). Time constant should be in the range of 4.8-5.1 ms (Bio-Rad Gene Pulser Xcell)
- After electroporation, add 1 ml LSLB, mix to recover the bacteria and transfer to a 15 ml Falcon tube. Incubate for 1 hour @32°C
- Transfer 250 µL of transformed bacteria to 5 mL LSLB in a 15 ml Falcon tube w/ cm (12.5 µg/ml) + hygromycin (100 µg/ml, to select for pSIM18). Incubate the tubes (one for each BAC) o/n @32°C (incubator)

Prepare cassette for co-targeting experiment

- Set up o/n cultures (50 µg/ml kan selection), 32°C incubator

Day 3

Prepare cassette for co-targeting experiment

- Miniprep analysis of the colonies. Test by restriction analysis with the enzyme used to flank your synthesis piece. You will see three bands (3 kb, approx. 2.2 kb, and 400-500 bp)
- Gel-purify the 2.2 kb targeting cassette *(synthesized cassette with homology arms, now containing Pgk1-em7-neo-pA resistance gene flanked by two FRT sites and a single loxP site).*

Co-targeting

- Dilute 250 µl of each of the two BACs containing pSIM18 into a single shaking flask containing 25 ml LSLB w/ cm+hyg. From this point there is only one BAC culture
- Incubate @32°C in shaking waterbath until culture reaches an OD_{600} ~0.55 (2.5-3 hours)
- Transfer flask to 42°C shaking waterbath for 15 min. It is important not to exceed 15 min.
- Proceed as in Box 1
- Transfer the pellet in the remaining volume to a cold microfuge tube. Spin 1 min at 10K rpm in table top centrifuge at 0°C. Carefully pipette off residual supernatant and resuspend in 100 µl ice-cold ddH_2O.
- In an eppendorf tube (on ice), mix 1 ng cassette with 1 µl 5 µM loxP oligo and 50 µl freshly prepared electrocompetent cells. Transfer to a 0.1 cm cuvette and electroporate. Use preset standard conditions for E. coli (1.8 kV, 25 µF, 200 Ω). Time constant should be in the range of 4.8-5.1 ms.
- After electroporation, add 1 ml LSLB, mix to recover the bacteria and transfer to a 15 ml Falcon tube. Incubate for 4 hours @32°C
- Aliquot 10 µL of transformed bacteria to each well except H12 (negative control) of a 96 deep well culture plate containing 500 µL of LSLB w/ kan (25 µg/ml). Incubate o/n @32°C (incubator), at least 24 hours.

Day 4

Co-targeting: Screen wells by PCR

- After at least 24 hours of growth, screen culture plate by PCR to detect shift of wt band by 34 bp
- 25 µl rxn, 27 cycles, using 3 µl of overnight culture. Screening primers should be positioned just outside of loxP oligo.
- Run PCR rxns on 1.5% gel. You will have wt bands in every well, look for the slightly larger band, most likely with lower intensity than wt band
- Combine 3-5 positive wells (a total of 500 µl) into 5 ml LSLB and incubate o/n @32°C w/kan (25 µg/ml), hyg (100 µg/ml) and cm (12.5 µg/ml)

Day 5

Retrieval

- Add 500 µl of combined positive well o/n culture into 25 ml LSLB w/kan (25 µg/ml), hyg (100 µg/ml) and cm (12.5 µg/ml) in a shaking flask
- Incubate @32°C until culture reaches an OD_{600} ~0.55 (2.5-3 hours)
- Transfer flask to 42°C waterbath for 15 min. It's important not to exceed 15 min.
- Proceed as in Box 1

- Transfer the pellet in the remaining volume to a cold microfuge tube. Spin 1 min at 10K rpm in table top centrifuge at 0°C. Carefully pipette off residual supernatant and resuspend in 100 µl of ice-cold ddH_2O.
- Transform 50 µl electrocompetent and heatshocked cells with 2.5 µl (20-50 ng) gel-purified linearized retrieval vector. Use preset standard conditions for E. coli (1.8 kV, 25 µF, 200 Ω). Time constant should be in the range of 4.8-5.1 ms.
- Recover for 1 hour @32°C in 1 ml LB (waterbath)
- Plate 100 µl and 200 µl on LB + 50 µg/ml carb plates. There is no need for kan selection as all the bacteria will be kan-resistant due to the presence of the BAC.
- Incubate plates **@37°C** o/n *(this is to cure the bacteria of the pSIM18 plasmid which has a temp. sensitive replication origin)*

Day 6

Analyze colonies for retrieval

- Set up o/n cultures (50 µg/ml carb + 50 µg/ml kan selection), **37°C** incubator

Day 7

Analyze colonies for retrieval

- Miniprep analysis of the colonies. Test by restriction analysis. Choose one or two enzymes that clearly show if you retrieved the correct fragment.
- Submit for sequencing with loxP check primers to confirm presence of loxP and that there are no errors resulting from the loxP oligo used in CoSBR.

Preparation of pre-made recombineering-ready SW102 cells:

- Using a sterile toothpick and SW102 cells from a glycerol stock, inoculate 5 ml LSLB w/ 12.5 µg/ml tetracycline
- Incubate o/n @32°C
- Dilute 500 µl of the o/n culture into a single flask containing 25 ml LSLB w/ 12.5 µg/ml tet.
- Prepare an ice-slurry (ice + water) in an ice bucket. Cool 25 ml 10% v/v glycerol in ddH_2O on ice.
- Incubate @32°C until culture reaches an OD_{600} ~0.55 (2.5-3 hours)
- Transfer flask to 42°C shaking waterbath for 15 min. Important not to exceed 15 min.
- Put the culture in the ice-slurry for 5 min. to cool.
- Spin down 10 ml of culture @ 2,800 x g for 3-5 min. in a pre-cooled centrifuge, @0°C.
- Pour off ALL the supernatant, gently tap on a papertowel to remove the last bit, and place the tube in the ice bucket.
- Add 1 ml ice-cold glycerol solution and, keeping it on ice, gently swirl the tube to resuspend the pellet.
- When pellet is completely resuspended, add 9 ml ice-cold glycerol solution, close the cap, and invert a couple of times, then spin again for 3-5 min.
- Repeat the washing step and spin one last time.
- After the final spin the pellet is very loose, so be careful when removing the supernatant the last time. *You should wait at the centrifuge to the last spin is completed, or you might not get there in time to avoid losing the pellet... Try to pour away all the supernatant while keeping an eye on the pellet while it starts sliding down the side of the tube.*
- Transfer the pellet in the remaining volume to a cold microfuge tube. Spin 1 min at 10K rpm in table top centrifuge at 0°C. Carefully pipette off residual supernatant and resuspend in 100 µl of ice-cold glycerol solution.
- Freeze 4 aliquots of 25 µl on dry ice and transfer to -80°C. for storage
- Use one aliquot for generation of the targeting cassette.
- **Note**: protocol can be scaled up, but heat-shock is most efficient in volumes up to 100 ml (16 tubes of 25 µl).

Box 2

463 bp targeting cassette template for synthesis ordering.

Simply insert gene-specific homology arms into the sequence below. BamHI sites are used below since BamHI doesn't cut the 1.9 kb insert from PL452, but any unique enzyme can be used.

5'-ggatcc-100 bp gene-specific homology arm upstream of FRT-gaagttcctattctctagaaagtataggaacttcggtctgaagaggagtttacgtccagccaagctagcttggctgcaggtcgtcgaaattctaccgggtaggggctctatggcttctgaggcggaaagaaccagctggggctcgactagagcttgcggaacccgaagttcctattctctagaaagtataggaacttcatcagtcaggtacataatataacttcgtataatgtatgctatacgaagttat-100 bp homology arm downstream of loxP-ggatcc-3'

Key:

Red: FRT site
Blue: homology to *Pgk1* promoter = 5'end of 1.9 kb fragment from PL452
Green: homology to BGH pA signal = 3'end of 1.9 kb fragment from PL452
Grey: spacer region to separate FRT from loxP
Purple: loxP

Media:

Low salt LB (LSLB or Lennox):
10 g Tryptone
5 g Yeast Extract
5 g NaCl
QS with ddH_2O to 1L
Autoclave

Low salt LB Agar:
20 g Lennox
15 g granulated agar
QS with ddH_2O to 1L
Autoclave
Cool, add antibiotic

Glycerol

Stock solutions for antibiotics (working dilution):

Kanamycin (kan): 50 mg/mL (1:1,000, 50 μg/ml)
Carbenicillin (carb): 50 mg/mL (1:1,000, 50 μg/ml)
Hygromycin (hyg): 100 mg/mL (HEPES) (1:1,000, 100 μg/ml)
Chloramphenicol (cm): 25 mg/mL (Ethanol) (1:2,000, 12.5 μg/ml)
Tetracycline (tet): 5 mg/ml (Ethanol) (1:400, 12.5 μg/ml)

Plasmids and SW102 cells

All recombineering reagents described in this study (SW102 cells plus PL452 and pSIM18 plasmids) were obtained from NCI (http://ncifrederick.cancer.gov/research/brb/recombineeringInformation.aspx.)

pBlightTK is available from us upon request.

Equipment

32°C incubator with 96 well plate shaker
32°C shaking waterbath (*e.g.* New Brunswick Scientific)
42°C shaking waterbath (*e.g.* New Brunswick Scientific)
Refrigerated centrifuge for 15 ml Falcon tubes
Baffled shaking flasks 50 ml with lids
